# Effects of a Single Yoga Session on Cardiac Interoceptive Accuracy and Emotional Experience

**DOI:** 10.3390/brainsci11121572

**Published:** 2021-11-28

**Authors:** Christine Schillings, Dana Schultchen, Olga Pollatos

**Affiliations:** Clinical and Health Psychology, Institute of Psychology and Education, Ulm University, 89069 Ulm, Germany; christine.schillings@uni-ulm.de (C.S.); olga.pollatos@uni-ulm.de (O.P.)

**Keywords:** interoception, cardiac interoceptive accuracy, emotional experience, affect, yoga

## Abstract

Background: There is an increasing body of research supporting the idea that cardiac interoceptive accuracy (IAc) can be improved by training. Findings concerning the effects of a single yoga session on IAc and the related construct emotional experience are sparse. The aim of this study was to examine if a single yoga session increases IAc and improves emotional experience. Methods: 137 students were randomly assigned to a 20-min yoga session (*n* = 47), an endurance session (*n* = 46), or an inactive control condition (*n* = 44). IAc and emotional experience were assessed before and after the sessions. Results: There were no significant changes in IAc, or positive and negative affect. IAc at baseline and the change in positive effect were found as predictors for IAc after the yoga session. Conclusion: A 20-min yoga session seems to be not applicable to improve IAc and emotional experience. Future studies should investigate long-term interventions and diverse healthy and clinical populations.

## 1. Introduction

### 1.1. Interoception

Interoception is defined as the process by which the nervous system senses, interprets, and integrates internal bodily signals to form a moment-by-moment internal bodily landscape across conscious and unconscious levels [1]. Individuals with better interoceptive abilities have been shown to experience emotions more intensely and are better able to regulate emotions in situations related to internal bodily changes like increased respiration or heart rate (e.g., [2,3,4,5,6,7,8]). This is in accordance with several emotional theories [9,10,11,12] postulating that perceiving internal bodily signals is essential for emotional experience.

Garfinkel and colleagues [13] proposed different facets of interoceptive abilities, namely, interoceptive accuracy (IAc), sensibility, and awareness. IAc represents the objective accuracy in detecting signals from within the body, mostly assessed via behavioral performance measures like the heartbeat perception task [14]. Impaired IAc has been found in various mental disorders such as depression [15,16], anorexia nervosa [17,18], obsessive-compulsive disorder [19] and alexithymia [20,21]. Based on these results, it is necessary to improve interoceptive abilities in specific populations like individuals with such mental disorders.

### 1.2. The Association between Cardiac Interoceptive Accuracy and Physical Activity

Previous studies [22,23,24,25,26] have investigated the predictive association between IAc and physical activity as well as the effect of physical activity on IAc. First evidence showing a positive association between IAc and physical activity was found in a study with long-distance runners who exhibited higher IAc compared to participants in a sedentary group and those with low levels of physical fitness [25]. A study by Herbert and colleagues [26] showed that higher IAc is associated with better behavioral self-regulation of physical load. Thereby, good heartbeat perceivers covered a shorter distance, had a smaller increase in mean heart rate, stroke volume, and cardiac output than poor heartbeat perceivers when cycling on a bicycle ergometer. Similarly, children with higher IAc performed better in a running performance task [24]. In contrast, Montgomery and Jones [23] showed that only participants with a moderate physical activity level exhibited heightened IAc during exercise. Köteles and colleagues [22] compared IAc under resting and physical load conditions and found that IAc at rest was associated with the ability to reproduce the heartrate under a resting and physical load only for very low load but not for higher loads. 

### 1.3. Approaches to Train Cardiac Interoceptive Accuracy

There is an increasing body of research supporting the idea that IAc can be trained. Different approaches focus on self-focused procedures increasing attention to the internal body via a photo of one-self, the own mirror image, or self-relevant words [27,28,29,30,31], body-focused training, such as power posing [32], or cardiac biofeedback training [33,34]. Other approaches that have been employed to increase interoceptive abilities are mindfulness-based interventions, where participants practice sustaining attention to internal bodily sensations, such as heartbeat, breath, or muscle tension. Such attention-focused methods have been shown to result in enhanced awareness of physical perceptions, sensations, and affective states [35,36,37].

Up to now, findings regarding the effects of mindfulness-based interventions on IAc are incongruent. In a study by Fischer and colleagues [38], participants exhibited higher IAc after an 8-week body scan intervention compared to a control group listening to an audiobook. Similarly, in a study by Bornemann and Singer [3], increased IAc was found due to a 9-month mental and interoceptive training program, compared to a 3-month mental training program and a test-retest control group. In contrast, Parkin and colleagues [39] did not find improvements in IAc due to different mindfulness-based interventions. Results of a study by Fairclough and Goodwin [40] also showed no increase in IAc due to two meditation sessions focusing on a yoga breathing technique. Similarly, meditators and non-meditators did not differ in their IAc performance [36,41,42]. A recent study by Demartini and colleagues [43] found that a single yoga class did not improve IAc in patients with anorexia nervosa, whereas IAc led to improvements in IAc of healthy control participants. In conclusion, the described studies differ in their mindfulness intervention types and duration, which could explain the various results. 

### 1.4. The Effect of Yoga Practice on Health-Related Parameters

Besides the effects of mindfulness-based interventions on IAc, the effects of yoga practice on further health-relevant variables should be considered. Previous studies showed promising effects on psychological and physiological parameters due to yoga interventions, among diverse healthy and clinical samples, such as increased positive affect and well-being (e.g., [44,45,46,47]), positive effects on brain structures, such as the hippocampus, the amygdala, the prefrontal, and the cingulate cortex in terms of functional brain connectivity [48], decreased heartrate and blood pressure (e.g., [49,50,51]), stress reduction (e.g., [52,53]), reduced symptoms of anxiety (e.g., [54,55]), depression (e.g., [56,57]), and pain syndromes (e.g., [58,59]). More generally, yoga practice has the potential to increase embodiment (i.e., body awareness and body responsiveness), by, for example, encouraging practitioners to listen and accept their body sensations [60]. A framework by Gard et al. [61] describes diverse self-regulatory mechanisms of yoga on a cognitive, emotional, behavioral, and autonomic level.

### 1.5. The Effect of Yoga and Physical Exercises on Emotional Experience

Furthermore, emotional experience–as linked to interoceptive abilities–should be considered. Positive affect refers to positive emotions, such as happiness, calmness, and, negative affect, respectively, describes negative affective states, such as sadness or fear (e.g., [62,63]). Significant increases in positive affect and decreases in negative affect were found due to yoga interventions in diverse samples, e.g., in healthy participants due to a one-week yoga camp [64], in yoga practitioners due to an 8-week yoga training [60], or in breast cancer patients due to a 6-week yoga training [65]. Even after a single yoga session, increased positive affect and reduced negative affect were found in a sample of public school students [66] and college women [67]. In contrast, comparable studies only found decreases in negative affect, but no improvements in positive affect due to a single yoga session [68,69] or due to a 10-week yoga intervention in female students [70]. Focusing on endurance or aerobic exercise, meta-analyses have indicated that there are improvements in emotional experience from single sessions, in both healthy and clinical samples (e.g., [71,72]). Comparable results on positive affect were found in a review by Liao and colleagues [73], whereas evidence for reduced negative affect was inconsistent. A review based on studies comparing the effects of yoga and endurance exercise [74] showed that, in both healthy and clinical populations, yoga was as effective as or better than exercise in improving diverse health-related parameters, such as stress levels or heartrate. 

### 1.6. Aims and Hypotheses of the Present Study 

Besides a broad range of positive physiological and psychological effects, yoga seems to be a promising approach to improve IAc due to its special body- and attention-related focus. To sum up, there is evidence to suggest that training paradigms to improve IAc have important ramifications for health-related parameters. Further research on this is necessary because previous results concerning the effect of mindfulness-based interventions on IAc and emotional experience are incongruent and evidence concerning the effects of a single session of yoga and endurance exercise is sparse. Moreover, we considered and compared two different 20-min sessions of physical exercises–yoga and endurance (stepper) exercises–to better understand the effects on interoception. In our opinion, yoga tends to include additional mindful attention towards the body whereas endurance exercise does not. Moreover, and to the best of our knowledge, there is no study investigating the effects of yoga and endurance sessions on both IAc and emotional experience and health- as well as interoception-related control variables. Additionally, both active groups were compared to an inactive control group. 

As the first hypothesis, an increase in IAc in the yoga group and in the endurance group was assumed. Because of the body- and attention-related focus of yoga, we expected a higher increase in the yoga group. Secondly, an increase in positive affect and a greater reduction in negative affect with greater improvements for the yoga group were hypothesized. Thirdly, based on the relationship of IAc and emotional experience and the body- and attention-related focus of yoga, we examined if IAc after the yoga intervention was predicted by IAc at baseline, changes in positive and negative affect pre- to post-intervention, and different health- and interoception-related predictor variables, such as sex, BMI, and physical activity level. The different predictors were chosen based on the previous literature indicating associations between interoception and health-related parameters, such as sex (e.g., [74,75,76]), BMI (e.g., [77,78]) and cardiovascular factors (e.g., [22,79]).

## 2. Materials and Methods

### 2.1. Participants

To calculate the sample size, an a-priori power analysis for a repeated measurement ANOVA via G*Power [80] was conducted. Assuming a small effect size of f = 0.15 (consistent with d = 0.30), based on a power of 0.90 and an α-level of 0.05, the analysis resulted in a sample size of *N* = 132. Considering potential dropouts, a total number of 137 students with a mean age of 23.7 (SD = 4.0) were recruited at Ulm University using announcements and mailing lists. Participants were randomly assigned to the yoga (*n* = 47), the endurance group (*n* = 46), or the inactive control group (*n* = 44). An exclusion criterion was injuries because of possible physical constraints during the exercises. The yoga group included 30 women and 17 men (M_Age_ = 24.11; SD_Age_ = 4.44), the endurance group consisted of 29 women and 17 men (M_Age_ = 23.89; SD_Age_ = 4.02), while the inactive control group included 34 women and 10 men (M_Age_ = 23.09; SD_Age_ = 3.32).

### 2.2. Procedure and Materials

Initially, participants were informed about the purpose of the study at a laboratory at Ulm University. After reading and signing the informed consent, participants completed a questionnaire on sociodemographic variables (e.g., age, sex, education) via the online survey platform LimeSurvey 2.5. Subsequently, participants were randomly assigned to the groups. After weight and height had been assessed, they completed the Freiburg Questionnaire of Physical Activity [81] and the German version of the Positive and Negative Affect Schedule (PANAS; [82]) as paper-pencil questionnaires. Furthermore, heartrate was assessed during the performance of the heartbeat perception task [14] using the mobile heart frequency monitor RS800CX (Polar Electro Oy, Kempele, Finland). After the 20-min yoga or endurance session, respectively, 20 min of inactivity, participants performed the heartbeat perception task and the PANAS again. After the participants had successfully completed all parts of the study, they received 1.5 credit points or 10 euros. The questionnaires and procedures used in this study will be explained in more detail in the following.

### 2.3. Emotional Experience

The German PANAS by Krohne and colleagues [82] is a frequently used questionnaire to measure emotional experience, more specifically, positive and negative affect. Participants had to rate their actual emotional states on a Likert scale ranging from 1 (=not at all) to 5 (=extremely). The questionnaire comprises two subscales, each including 10 items representing positive affect and negative affect. Whereas the positive affect subscale includes items like “active” and “strong”, the negative subscale comprises items like “nervous” and “upset”. For each of the two subscales, a total score was calculated. Higher scores on the positive affect scale reflect a state of “full of energy, high concentration and pleasurable engagement”. In contrast, higher scores on the negative affect scale indicate more distress. Similar to internal consistency scores from a previous study [62] for positive affect (Cronbach’s α = 0.86 to 0.90) and negative affect (Cronbach’s α = 0.84 to 0.87), in the present study, Cronbach’s α for positive affect was 0.84 at pre- and 0.90 at post-measurement. For negative affect, Cronbach’s α was 0.89 at pre- and 0.90 at post-measurement.

### 2.4. Physical Activity

To assess physical activity and the corresponding physical activity levels in daily life, the short form of the Freiburg Questionnaire of Physical Activity by Frey et al. [81] was used. This questionnaire assesses occupational, household, and leisure activities (e.g., walking, cycling, climbing stairs), as well as sports activities of the past week, or, respectively, of the past month. The participants’ overall activity levels based on the time of all activities per week were converted into metabolic equivalent (MET) values, representing the ratio of a metabolic rate during physical activity to a resting metabolic rate. The standardized values of the MET originate from an accordant compendium [83]. Moreover, we categorized the physical activity levels into three groups, namely low (<30 MET), moderately (30–60 MET), and highly active (≥60 MET), as Frey and colleagues [84] suggested.

### 2.5. Cardiac Interoceptive Accuracy (IAc)

Cardiac interoceptive accuracy was assessed via the heartbeat perception task [14]. The participants were instructed to focus on their heartbeats and to count them silently during three different time intervals (lengths: 25, 35, 45 s), without taking their pulse or using other manipulating strategies (e.g., stop breathing). Importantly, the participants should count only those heartbeats of which they were sure of [85]. Moreover, they should sit in a relaxed position, stop any movements, and, if preferred, close their eyes during the task. They neither had information about the lengths of the intervals nor did they get feedback about their performance. To get familiar with the task, participants had to perform a 10-s training interval. If they had no more questions, the first interval started. Every interval was introduced with a start signal and ended with a stop signal given by the instructor. During the 20-s break between the intervals, the participants had to report their counted heartbeats. At the same time, the recorded heartbeats were measured via a polar watch (RS800CX; Polar Electro Oy, Kempele, Finland). The advantages of this method are that it is mobile, easy to use, non-invasive, and also recommended for field studies [77]. Moreover, the validity and reliability related to comparable ECG devices have been demonstrated in different studies (e.g., [86,87]). Cardiovascular data were analyzed using the software “Polar Pro Trainer 5” (Version 5.40.172). Lastly, the averaged heartbeat perception scores—representing the IAc scores—were calculated according to the following formula:

$$\mathrm{IAc Score}=\frac{1}{3}\;\sum (1-\frac{\left(\left|\mathrm{recorded}\;\mathrm{heartbeats}-\mathrm{counted}\;\mathrm{heartbeats}\right|\right)}{\mathrm{recorded}\;\mathrm{heartbeats}}$$

The calculated IAc score varies between 0 and 1, higher scores represent higher IAc, indicating a higher sensitivity for cardiovascular signals [88].

### 2.6. Group: Yoga, Endurance, and Inactive Control Group

One of the two investigators instructed the standardized activity sessions, performing them individually with each participant at a time. The two instructors who were familiar with yoga constructed the yoga session consisting of cultural asanas and had rehearsed it together to guarantee a high level of standardization. Both activity programs lasted about 20 min. Before the yoga or the endurance exercises, a warm-up phase consisting of two minutes of jumping was conducted. The focus of the different yoga exercises was on flexibility, coordination, and strength. The 20-min yoga session consisted of two rounds of six consecutively performed instructed asanas (e.g., Warrior I, Chair, Cobra and Dolphin Pose). For the endurance session, participants had to perform two rounds of nine different stepper exercises (each one lasting one minute). Participants in the control group were asked to sit still in a quiet, isolated room for 20 min and were not offered any structured activities.

### 2.7. Data Analysis

For the data analyses, the Statistical Package for Social Sciences version 26.0 was used. Firstly, descriptive data to summarize the data and a one-way ANOVA to test for group differences in the baseline data were calculated. Moreover, a GLM repeated measures ANOVA was conducted with the within-subjects factor of both measurements (IAc as well as positive/negative affect before and after the session) and between-subjects factors of group with three levels (yoga, endurance, or inactive control group). A GLM repeated measures ANCOVA with the control variable heartrate after the sessions was also calculated. Effect sizes η_p_^2^ for repeated measures ANOVAs and the ANCOVA as well as effect size were reported. A forward stepping regression analysis with IAc after the yoga session as the criterion variable and different predictor variables (IAc at baseline, negative affect at baseline, differences in positive and negative affect, sex, BMI, physical activity level) was calculated.

## 3. Results

### 3.1. Descriptive Statistics

There were no significant differences between the groups regarding the participants’ age, BMI, sex, physical activity levels, and IAc, but there were significant differences for negative affect at baseline. Therefore, negative affect at baseline was calculated as a control variable in the regression analysis. For a summary of the descriptive statistics, see Table 1 and Table 2. Furthermore, there were no significant differences in the baselines heartrate before the sessions of yoga (M = 73.96; SD = 12.11) and stepper exercise (M = 76.52; SD = 13.03); F (1, 91) = 0.97, *p* = 0.328.

### 3.2. Changes in Cardiac Interoceptive Accuracy

**Figure 1:**
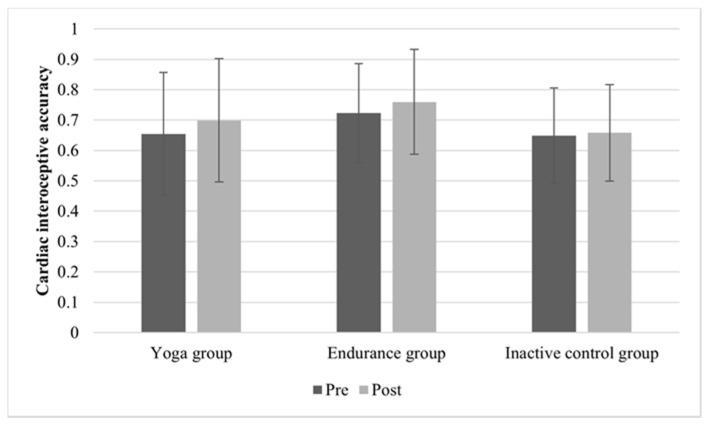
Mean (SD) cardiac interoceptive accuracy before and after 20-min of yoga, endurance, or non-activity.

The mean values and standard deviations are depicted in Figure 1. On the descriptive level, all three groups exhibited small increases in IAc (yoga group: M_Pre_ = 0.66, SD_Pre_ = 0.20; M_Post_ = 0.70, SD_Post_ = 0.20; endurance group: M_Pre_ = 0.72, SD_Pre_ = 0.16; M_Post_ = 0.76, SD_Post_ = 0.17; control group: M_Pre_ = 0.65, SD_Pre_ = 0.16; M_Post_ = 0.66, SD_Post_ = 0.16). The ANOVA analysis revealed significant main effects of time (F (1, 133) = 3.98; *p* = 0.048; η_p_^2^ = 0.029) and group (F (2, 133) = 3.97; *p* = 0.021; η_p_^2^ = 0.056), indicating differences due to the conditions and in the groups. The time x group interaction (F (2, 133) = 1.08; *p* = 0.345; η_p_^2^ = 0.016) was not significant, demonstrating that IAc did not change over time depending on the group.

The ANCOVA analyses revealed that the control variable mean heart rate during physical activity (yoga/stepper) did not significantly influence IAc (F (1, 88) = 1.17; *p* = 0.284; η_p_^2^ = 0.013). The control variable heartrate after the sessions significantly influenced the change in IAc (F (1, 132) = 5.94; *p* = 0.016, η_p_^2^ = 0.043), but did not interact with the time effect (F (1, 132) = 0.062; *p* = 0.804). Furthermore, the mean change in IAc and the mean change in the heartrate from before to after the sessions was uncorrelated for the yoga group (mean change IAc = 0.04; SD = 0.13; mean change heartrate = 14.33; SD = 11.21; r = -0.175, *p* = 0.12, one-tailed) and for the endurance group (mean change IAc = 0.04; SD = 0.19; mean change heartrate = 18.20; SD = 12.11; r = 0.095, *p* = 0.534). The heartrate during the activity in the yoga group (M = 117.43, SD = 15.39) differed significantly from the one in the endurance group (M = 131.78, SD = 15.67; t (90) = 4.432; *p* = 0.001). The control variables sex, BMI, physical activity level, and negative affect at baseline did not significantly influence IAc.

### 3.3. Changes in Emotional Experience

**Figure 2:**
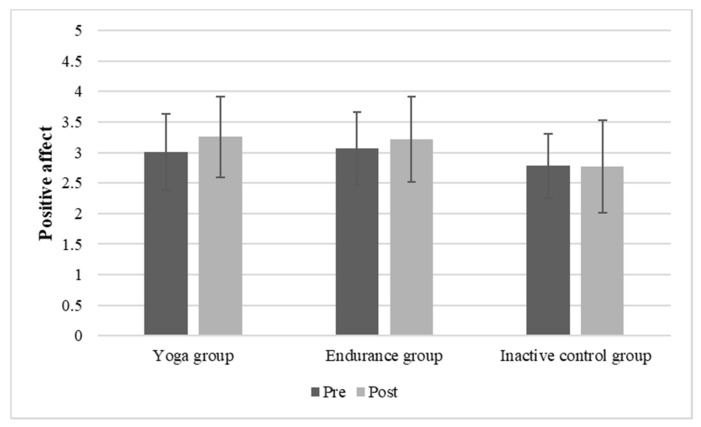
Mean (SD) positive affect before and after 20-min of yoga, endurance, or non-activity.

Regarding positive affect, significant main effects of time (F (1, 132) = 8.14; *p* = 0.005; η_p_^2^ = 0.058) and group (F (2, 132) = 5.516; *p* = 0.005; η_p_^2^ = 0.077) were found, representing a change in positive affect due to the conditions and differences in the groups. Regarding the interaction time x group, no significant differences were found (F (2, 132) = 2.81; *p* = 0.064). There was also no significant increase of the yoga group only compared to the endurance group (*p* = 0.177; r_effect size_ = 0.079). All mean values and standard deviations regarding positive affect of the different groups are depicted in Figure 2.

**Figure 3:**
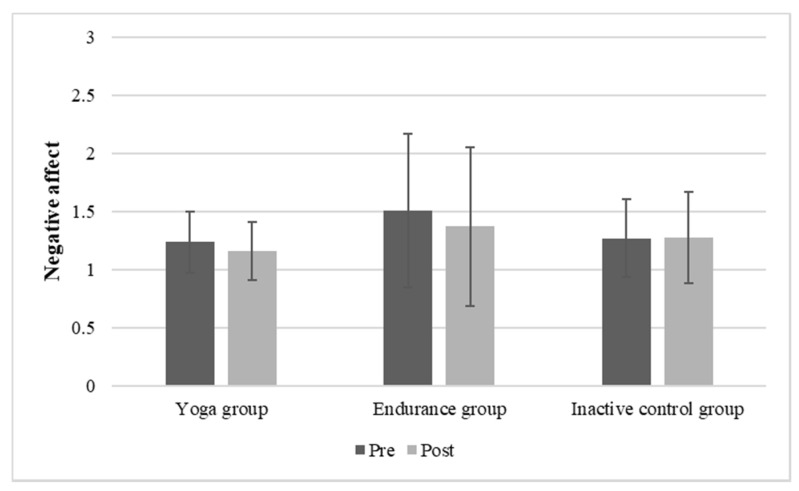
Mean (SD) negative affect before and after 20-min of yoga, endurance, or non-activity.

Concerning negative affect, it should be noted that there were significant differences at the baseline measurement concerning the different groups (see Table 1). However, the main effect of time (F (1, 132) = 7.52; *p* = 0.007; η_p_^2^ = 0.054) and group were significant (F (1, 132) = 3.51; *p* = 0.033; η_p_^2^ = 0.038), whereas there was no significant time x group interaction (F (1, 132) = 2.58; *p* = 0.079; η_p_^2^ = 0.050). The mean values and standard deviations for negative affect of each group are depicted in Figure 3.

A multiple regression analysis was calculated for the yoga group. Using a forward stepping regression analysis with IAc after yoga as the criterion variable, the following variables were tested as predictors: IAc at baseline, sex, BMI, physical activity level, negative affect at baseline, change in positive affect, and change in negative affect. Apart from a small correlation of IAc at baseline and BMI (r = 0.292, *p* = 0.046) as well as sex and BMI (r = 0.41, *p* < 0.005), no significant associations of the predictor variables for the yoga group were found. Results show that the final model including only IAc at baseline and the change in positive affect as significant predictors (F (2, 45) = 46.54, *p* < 0.001) explained 67% (corrected R^2^) of the total variance (see Table 3). Consequently, according to this model, IAc at baseline as well as the increase in positive affect were found as significant predictors of IAc after the yoga session, demonstrating that IAc at baseline and the enhanced positive affect influenced the IAc score after the yoga activity. Sex, BMI, physical activity level, negative affect at baseline and the change in negative affect had no influence on IAc after yoga. All regression coefficients of the final model are depicted in Table 4.

## 4. Discussion

This study aimed to examine if a 20-min yoga session improves IAc and emotional experience compared to an endurance session and an inactive control group. We could not find any effects of a single yoga session on IAc, or positive and negative affect. Nevertheless, IAc at baseline as well as the change in positive affect predicted IAc after the yoga session. Sex, BMI, physical activity, level, negative affect at baseline, and the change in negative affect did not predict IAc after the yoga session.

### 4.1. Effect of Yoga on Cardiac Interoceptive Accuracy

As we did not observe a significant increase in IAc in either of the active groups, this hypothesis was not supported by our data. The effect of yoga on IAc failing to reach significance might be explained by the potential mechanism that after single sessions of yoga, the participants could not engage in the self- and body-focus which several sessions of yoga or other self- and body-focused training procedures might evoke. In contrast to our results, several previous studies have shown enhanced IAc due to diverse short-term manipulation techniques of self-focused training [27,28,29,30,31,32,33]. Accordingly, improved IAc could be explained by the increase in the attentional focus on the body [35,36,37]. This focus might be only developed over several yoga sessions, depending upon an individual’s ability to focus on oneself and familiarization with yoga sessions. According to this approach, for example, increased IAc could be found due to mindfulness-based training after practicing mindfulness for at least eight weeks [3,38].

Contrary to the present results are also the findings by Demartini and colleagues [43] which showed improvements in IAc in healthy participants due to a single yoga class. It needs to be noted that in this pilot study, the participants were compared to patients with anorexia nervosa who exhibited significantly lower IAc before and after the yoga session, which also lasted 75 min, as compared to our 20-min session.

Another possible explanation for our missing effects might refer to the sample composition consisting of students and more female than male participants. Particularly, men tend to exhibit a higher IAc than women [76,89]. To analyze the influence of such characteristics in more detail, further healthy and clinical gender-balanced samples are needed. Furthermore, the level of previous experience with yoga or mindfulness could have been an influencing variable.

### 4.2. Effect of Yoga vs. Endurance Exercise on Emotional Experience

We expected that physical exercise conditions would both increase positive affect and decrease negative affect, with a greater change in the yoga group than the endurance group. These changes were not supported by our data. The results could not support our assumption of mood improvements due to both activity programs. The missing improvements in positive and negative affect are in contrast to previous findings concerning a single yoga session [66,67] and even long-term yoga training [60,64,65], which also used the PANAS, as well as findings showing decreased negative affect due to a single yoga session [68,69]. In line with the present study, these studies found no decrease in positive affect. It should be noted that the presented results concerning negative affect need to be interpreted carefully due to the significant differences for negative affect at baseline, which we controlled for in the statistical analyses. The missing effects of the single yoga session on improvements in positive and negative affect might be explained by the length of the session which might need to be longer. Furthermore, future studies should compare the effects of diverse types of yoga that might differ in their potential to evoke significant mood improvements due to a single session. 

### 4.3. Change in Positive Affect as a Predictor for IAc in the Yoga Group

The present study provides the first evidence that IAc after a yoga session is also predicted by the change in positive affect. This finding needs to be interpreted carefully, as IAc did not significantly change due to the yoga session. On the basis of previous studies showing a positive relationship between emotional experience and IAc (e.g., [2,3,4,5,7,8,20], we assume that bodily changes and their perception are essential information for emotional experience. A neurophysiological explanation might be based on the link between emotional experience and bodily representations via a “convergence zone” in the anterior insula [90]. Speculating about the neurophysiological level, the anterior insula region, and the dorsomedial prefrontal cortex might underlie both interoceptive abilities [5,91,92] as well as sensory processing during mindfulness practice [93,94] and, could, therefore, act as an interface region activated via the yoga session. Furthermore, previous studies showed that high IAc was related to better self-regulation [26,95,96,97,98]. At the same time, yoga can enhance self-regulation, exemplarily, via increased meta-awareness and, more generally, integrated top-down and bottom-up mechanisms for self-regulation [61]. According to the systems network model supposed by these authors, meta-awareness of interoceptive processes enables the respective afferent and re-afferent input, such as somatosensory or viscerosensory processes as well as the integration and bidirectional feedback across high- and low-level networks. Integrating also the findings [99] showing that self-regulation mediated mindfulness and positive affect, self-regulation might be a contributing factor in the effect mechanisms of yoga on positive affect and IAc and the effect of the change in positive affect on IAc. Nevertheless, future research also integrating neurophysiological correlates is needed to prove this explanatory approach.

### 4.4. Limitations and Future Research

Some limitations of the present study should be declared. Firstly, only one dimension of interoception, namely IAc, was assessed. According to the three-dimensional model [13], interoceptive sensibility represents subjectively reported data concerning one’s own ability to focus on internal bodily sensations as assessed via questionnaires or confidence ratings during the heartbeat perception task. This could be an additional assessment for future studies. Secondly, yoga exercises are characterized by a high focus on breathing. Therefore, it could be of relevance to complement the study design by interoceptive tasks targeting the respiratory system as used by several research groups [3,100,101,102,103] and to compare specific effects of meditation and yoga focusing on breathing. Thirdly, we did not control for emotional experience directly related to the activity programs or previous experience with yoga.

Moreover, recent debates concerning the reliability and validity of the heartbeat perception task (e.g., [75,104,105,106,107,108]) should be considered. Nevertheless, contrary approaches [109,110] justify the reliability and the validity of the heartbeat perception task and, in the current study, the “strict instruction” meaning to count exclusively those heartbeats of which the participants actually perceived, was used. This instruction reduces the risk that the knowledge about the individual heartbeat influences IAc [111].

More generally, future research should investigate the long-term effects of yoga on IAc and emotional experience, including assessments of self-regulation and mindfulness to provide a complete explanatory approach including neurobiological and psychological factors. Furthermore, it would be interesting to assess the degree of attentional focus and to use experience sampling methods to measure emotional experience before and after the yoga session, e.g., via smartphones, which is proposed by Murphy and colleagues [112] and applied more and more (e.g., [113,114]). 

## 5. Conclusions

A 20-min yoga session seems to be not applicable to improve IAc and emotional experience. The present study provides the first evidence that IAc after the yoga session is predicted by the change in positive affect, which needs to be replicated in future research. Future studies should also investigate long-term interventions and different types of yoga. It could be of high interest to examine diverse healthy and clinical populations, especially samples with low interoceptive abilities.

## Figures and Tables

**Table 1:** Descriptive statistics at baseline per group.

	Yoga Group	Endurance Group	Control Group		
	Mean (SD)	Mean (SD)	Mean (SD)	F (2, 134)	*p*
Age	24.11 (4.44)	23.89 (4.02)	23.09 (3.33)	0.82	0.443
BMI	22.65 (3.12)	22.69 (4.23)	23.55 (5.06)	0.67	0.516
Positive Affect	3.01 (0.63)	3.07 (0.59)	2.78 (0.52)	3.05	0.051
Negative Affect	1.24 (0.27)	1.51 (0.66)	1.28 (0.33)	4.7	0.011
Cardiac IAc	0.65 (.20)	0.72 (0.16)	0.65 (0.16)	2.48	0.089

Note. SD, standard deviation; BMI, body mass index; IAc, interoceptive accuracy.

**Table 2:** Sex and physical activity levels at baseline per group.

	Yoga Group	Endurance Group	Control Group	df	Χ^2^	*p*
**Sex**				2	2.63	0.269
Male	17	17	10			
Female	30	29	34			
**Physical Activity level**				4	5.16	0.272
Low	12	16	14			
Moderate	13	17	18			
High	22	13	12			

**Table 3:** Model summary of the final regression model.

R	R^2^	Corr. R^2^	SE
0.827	0.684	0.669	0.118

Note. R, correlation between the observed and predicted values of the criterion variable; R^2^, explained variance; Corr. R^2^, corrected R^2^; SE, standard error.

**Table 4:** Final model of the forward-stepping multiple linear regression analysis (*n* = 47) with the predictors’ cardiac interoceptive accuracy at baseline, change in positive affect, and the criterion variable cardiac interoceptive accuracy after yoga.

Independent Variable	Standardized Beta	SE	t	*p*	95% CI Lower Limit	95% CI Upper Limit
Cardiac	0.797	0.086	9.287	<0.001	0.626	0.973
IAc (baseline)						
Change in positive affect	0.186	0.034	2.173	0.035	0.005	0.143

Note. SE, standard error; IAc, interoceptive accuracy; CI, confidence interval.

## Data Availability

The data presented in this study are available on request.
